# Bovine mastitis: Risk factors and isolation of Gram-negative bacteria in Western Algeria

**DOI:** 10.17221/40/2025-VETMED

**Published:** 2026-02-26

**Authors:** Cheimaa Bounoua, Djahida Souna, Mohammed El Amine Bekara, Ibrahim Belabdi, Mohammed Sebaihia, Nora Mimoune

**Affiliations:** ^1^Laboratory of Molecular Biology, Genomic and Bioinformatic, Department of Biology, Faculty of Nature and Life Sciences, University Hassiba Benbouali of Chlef, Chlef, Algeria; ^2^Department of Biology, Faculty of Nature and Life Sciences, University Hassiba Benbouali of Chlef, Chlef, Algeria; ^3^Higher National Veterinary School, Algiers, Algeria; Biotechnologies Platform for Animal Medicine & Reproduction (BIOMERA), Saad Dahleb Blida University 1, Blida, Algeria

**Keywords:** dairy herds, environmental effect, epidemiology, prevalence, subclinical and clinical mastitis

## Abstract

A cross-sectional study was conducted in western Algeria between February and October 2024, aimed at determining the prevalence of bovine mastitis, Gram-negative bacilli, and their associated risk factors. A total of 524 cows were sampled using clinical examination and the California mastitis test. Milk samples from the affected quarter were subjected to bacteriological assays. Three generalised linear mixed models were used to identify the risk factors for clinical mastitis (CM) and subclinical mastitis (SCM), as well as Gram-negative bacilli isolates associated with bovine mastitis. Model selection was performed using the Akaike information criterion. The prevalence of mastitis was 59.7% at the cow level, of which 12.6% was CM and 47.14% SCM. Overall, 65.5% of the mastitis cows showed a positive culture for Gram-negative bacilli. The most commonly isolated bacteria were *Escherichia* spp. (44%), *Klebsiella* spp. (23.1%), and *Pseudomonas* spp. (11.6%). The parity, contact with visitors, and daily milk yield (l/day) were identified as risk factors for SCM in dairy cows. However, foremilk discarding reduced the risk of developing SCM. A history of mastitis, udder injuries, udder and leg hygiene scores, and parity were identified as risk factors for CM. Nevertheless, none of the variables examined were risk factors for an udder infection by the Gram-negative bacilli isolates.

Algeria is considered one of the leading importers and consumers of milk worldwide. Milk is subsidised by the government to ensure accessibility and affordability for the general population. The amount of fluid milk produced locally is estimated to cover more than half of the annual consumption of 4.5 billion litres. Algeria imports powdered milk in bulk to meet domestic demand, placing a burden on the public treasury’s import budget (Wedzerai 2024). Currently, northwest Algeria is emerging as a significant dairy production region (USDA FAS 2023).

Bovine mastitis, a common problem on dairy farms, significantly impacts the health and productivity of dairy cattle, resulting in considerable economic losses to the dairy industry (Kebbal et al. 2024; Tong et al. 2025). According to the most recent Algerian studies, mastitis prevalence ranges from 41.66% (Hocine et al. 2021) to 54.75% (Saidani et al. 2024). This disease is related to farm management practices and the prevalence of various bacterial agents. As described by Cobirka et al. (2020), mastitis can be categorised into clinical (CM) and subclinical mastitis (SCM) based on different characteristics. CM is characterised by visible signs in the milk or udder, in contrast to SCM.

Over recent decades, while the proportion of infectious mastitis cases has declined significantly, the prevalence of environmental mastitis has increased (Emon et al. 2024). It is essential to recognise that Gram-positive bacteria remain important mastitis pathogens in Algeria. Conversely, Gram-negative bacterial species such as *Escherichia* spp., *Klebsiella* spp., and *Pseudomonas* spp., which are widely distributed on dairy farms, are potential zoonotic pathogens causing widespread infections in animals and humans, including sepsis, pneumonia, wound infections, and urinary tract inflammation (Tong et al. 2025).

Most previous studies in Algeria have focused on the mastitis prevalence and a limited number of associated risk factors. Currently, information on Gram-negative bacteria in bovine mastitis cases, particularly in northwest Algeria, remains scarce. Therefore, this study aimed to determine the overall prevalence of bovine mastitis, identify the Gram-negative bacilli involved, and investigate the associated risk factors.

## MATERIAL AND METHODS

### Ethical statement

This study was approved by the Ethics Committee of the Faculty of Natural and Life Sciences, Hassiba Benbouali University of Chlef, Algeria (07/02/2024, Ref. No. SO-02/2024).

### Study area, population, and farm type

**Figure 1:**
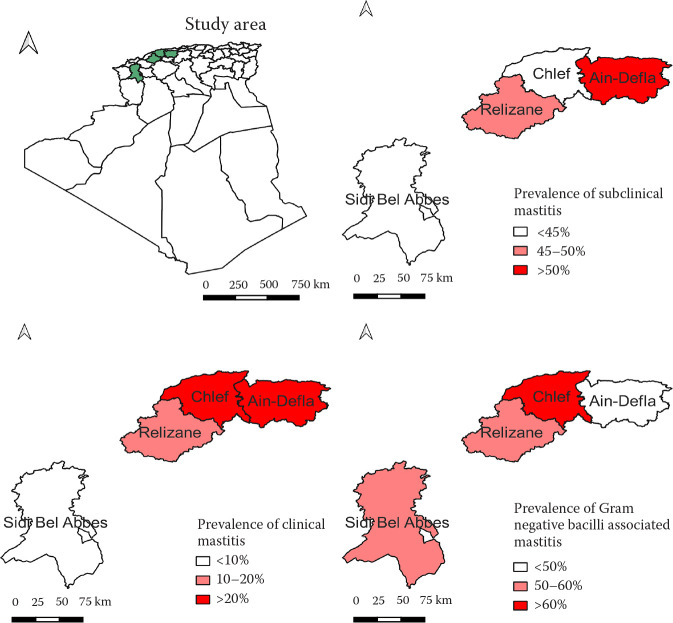
Geographical mapping of the study area showing the prevalence of clinical, subclinical and Gram-negative bacilli associated mastitis in northwest Algeria

A cross-sectional study was conducted between February and October 2024 in the northwest part of Algeria (Figure 1). A total of 34 farms located in the provinces of Chlef (*n*** = **5), Ain Defla (*n*** = **7), Relizane (*n*** = **8), and Sidi Bel Abbes (*n*** = **14) were randomly selected. The study included lactating cows raised for both beef and dairy production.

### Sample size and collection

The minimum expected number of lactating dairy cows to be included in the study was 384, calculated using the following formula (Thrusfield 2005):


$$n=\frac{Z_{\text{α}/2}^{2}\times P\times (1-P)}{d^{2}}$$ (1)

where:

*n* – sample size;

Z – percentile (1-alpha/2) of standard normal distribution;

α – set at 0.05 for 95% confidence interval = 1.96;

*P* – expected prevalence in population-based on previous studies;

*d* – desired absolute error or precision = 5%

With an expected prevalence (*P*) of 50%, at 5% desired absolute precision and a 95% confidence interval value of 1.96, each cow was randomly selected and examined for clinical signs related to the udder, teats, and visible milk changes. For cows with no clinical signs of mastitis, the California mastitis test (CMT) was performed on each quarter milk sample to detect SCM, as previously described by Abebe et al. (2016).

### Data collection

A structured survey was employed to collect data on the herd- and animal-level factors. The survey included questions on herd management, environment, hygiene, milking conditions, health status, previous mastitis history in the herd, and animal-related factors such as age, parity, and lactation stage.

### Isolation and identification of Gram-negative bacilli

Quarter milk samples were aseptically collected from CM and SCM cases following the procedure described by the National Mastitis Council (NMC) (Adkins et al. 2017). After the udder was washed and dried, and the teats were disinfected with 70% alcohol, the first streams of milk were discarded. Approximately 20 ml of milk was collected into sterile tubes and transported to the laboratory under cold conditions. Milk samples were inoculated into a Brain Heart Infusion broth and incubated at 37 °C. On the following day, a loopful of the enriched broth was cultured on Cetrimide agar and MacConkey agar. All the pure Gram-negative bacterial isolates were confirmed biochemically using the API 20E and API 20NE systems (bioMérieux, Lyon, France).

### Statistical analysis

The data were recorded in a Microsoft Excel spreadsheet and analysed using R software (v4.1.1) (R Core Team 2021). Descriptive measures, including percentages and absolute frequencies, were used to present and summarise the collected categorical data. A multivariable Generalised Linear Mixed Model (GLMM) with herd as a random effect was used to assess risk factors for SCM, CM, and Gram-negative bacilli-associated mastitis. The optimal model for each dependent variable was selected through a multi-step process: (1) A univariate analysis using GLMM was performed, and variables with *P* < 0.25 were retained, (2) a multicollinearity analysis was conducted among the variables selected from the univariate analysis. Variables with a variance inflation factor (VIF) <10 were then included for testing in a multivariable analysis. (3) A stepwise forward selection of the final multivariable model was carried out using the Akaike Information Criterion (AIC). The model with the lowest AIC value was chosen. The model’s overall goodness-of-fit was assessed using the Hosmer–Lemeshow test. The significance level was set at 5%. The results of the final model are presented as odds ratios with their 95% confidence intervals (95% CI).

## RESULTS

### Clinical, subclinical and Gram-negative bacilli-associated mastitis prevalence

Out of the 524 cows examined for the presence of mastitis, 59.7% showed evidence of mastitis. The prevalence of SCM was higher at 47.1% (95% CI: 42.8–51.4%) than that of CM at 12.6% (95% CI: 9.8–15.4%) at the cow level (Table 1). The highest prevalence of SCM was observed in the Ain Defla province (56.62%), followed by Relizane (47.79%), Sidi Bel Abbes (44.38%), and Chlef (41.28%). Conversely, the highest prevalence of CM was observed in Chlef (23.85%), followed by Ain Defla (21.68%), Relizane (13.23%), and Sidi Bel Abbes (4.08%) (Figure 1).

**Figure 2:**
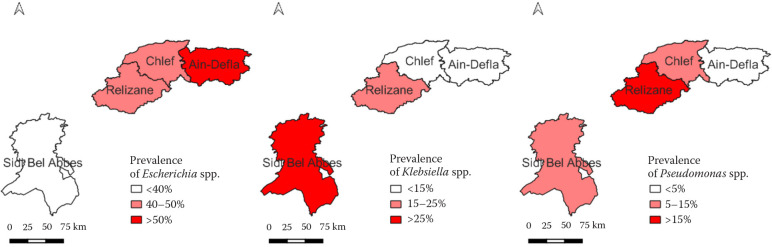
Prevalence of *Escherichia* spp., *Klebsiella* spp. and *Pseudomonas* spp*.* isolated from the milk samples in the Chlef, Ain Defla, Relizane and Sidi Bel Abbes provinces of Algeria

The prevalence of Gram-negative bacilli-associated mastitis was 65.5% (95% CI: 60.2–70.8%) at the cow level (Table 1). The most commonly isolated bacteria were *Escherichia* spp. (44%), *Klebsiella* spp. (23.1%), and *Pseudomonas* spp. (11.6%) (Figure 2).

**Table 1:** Prevalence of mastitis and Gram-negative bacilli-associated mastitis in northwest Algeria

Level	Number examined	Number positive for mastitis (%)		Total (%)	Number positive for Gram-negative bacilli associated mastitis (%)		Total (%)
		clinical	subclinical		clinical	subclinical	
Cow	524	66 (12.6%)	247 (47.1%)	313 (59.7%)	50 (75.7%)	155 (62.7%)	205 (65.5%)

### Risk factors analysis

Several herd- and cow-level factors were assessed for statistically significant associations with mastitis prevalence categories, including mastitis associated with Gram-negative bacilli, using univariate logistic regression [Electronic Supplementary Material (ESM) Table S1].

### Risk factors of subclinical mastitis

The results of the final model are presented in Table 2. Cows in their second (OR = 2.09; 95% CI: 1.25–3.29) and third (OR = 2.47; 95% CI: 1.31–4.17) parity had an increased risk of developing subclinical mastitis (SCM) compared to primiparous cows. However, cows with parity 4 or higher had a non-significantly higher risk than primiparous cows.

**Table 2:** Multivariable generalised linear mixed model analysis of the risk factors for subclinical mastitis (*n*** = **524)

Variables	Category	OR	Confidence interval at 95%	*P*-value*
Parity	1	reference		0.004
	2	2.09	1.25; 3.29	
	3	2.47	1.31; 4.17	
	≥4	1.69	0.84; 2.66	
				
Contact with visitors	no	reference		<0.001
	yes	3.50	1.79; 7.65	
				
Daily milk yield (l/d)	<5 l	reference		0.03
	5–10 l	2.48	0.56; 9.69	
	>10 l	5.71	1.65; 18.86	
				
Removal of the first jet of milk	no	reference		0.01
	yes	0.59	0.39; 0.89	

*P-value of variable effect

l/d** = **litres per day; OR** = **odds ratio

Furthermore, cows producing more than 10 litres of milk per day had a significantly higher likelihood of developing SCM compared to those producing less than 5 litres per day (OR = 5.71; 95% CI: 1.65–18.86).

Animals raised on farms with frequent visitor contact had a significantly increased risk of developing SCM compared to those on farms where visitor contact was limited (OR = 3.50; 95% CI: 1.79–7.65). Finally, cows whose farmers systematically discarded the foremilk before milking had a reduced risk of developing SCM (OR = 0.59; 95% CI: 0.39–0.89).

The Hosmer–Lemeshow goodness-of-fit test for the final model was considered acceptable (*P = *0.71).

### Risk factors of clinical mastitis

The results presented in Table 3 indicate that cows with a history of mastitis (OR = 2.93; 95% CI: 1.39–6.16) and those with udder injuries (OR = 9.08; 95% CI: 2.14–18.58) had a significantly higher risk of developing CM. Regarding parity, cows in their second parity had a lower risk of developing CM compared to primiparous cows (OR = 0.36; 95% CI: 0.19–0.95). Additionally, cows with dirtier udders and legs (score ≥ 3) were more likely to develop CM than those with clean udders and legs (score = 1).

**Table 3:** Multivariable generalised linear mixed model analysis of the risk factors for clinical mastitis (*n*** = **524)

Variables	Category	OR	Confidence interval at 95%	*P*-value*
Antecedents mastitis	no	reference		<0.001
	yes	2.93	1.39; 6.16	
				
Udder injuries	no	reference		0.004
	yes	9.08	2.14; 18.58	
				
Udder and leg hygiene score	1	reference		<0.001
	2	2.30	0.77; 6.85	
	3	9.28	3.34; 15.81	
	4	17.79	4.75; 35.76	
				
Parity	1	reference		0.04
	2	0.36	0.19; 0.95	
	3	0.56	0.32; 1.78	
	≥4	0.55	0.32; 1.76	

*P-value of variable effect

OR** = **odds ratio

The Hosmer–Lemeshow test for goodness-of-fit of the final model was considered acceptable (*P = *0.50).

### Risk factors of Gram-negative bacteria-associated mastitis

The final model indicated that none of the variables were associated with the prevalence of Gram-negative bacteria isolated from cows with mastitis.

## DISCUSSION

The study showed an overall mastitis prevalence of 59.7%. This prevalence closely aligns with findings reported in earlier studies (54.75% and 53%) by Saidani et al. (2024) and Abegewi et al. (2022). However, our finding is lower than the 72.8% prevalence reported by Zeleke and Demissie (2024) and higher than the 28.5% prevalence reported by Belachew and Fufa (2023). This might be due to several factors, including geographic region, sample size, diagnostic methods, management practices, or even cow-related factors.

The high prevalence of mastitis in this study could be explained by poor general management and inadequate mastitis prevention in the study areas, as only a small fraction of dairy herds maintained routine mastitis prevention practices. Additionally, managerial, epidemiological, environmental, and causative agents, along with the genetic make-up of the cows, may account for the prevalence variations observed in other studies (Saidani et al. 2024).

The prevalence of SCM was higher at 47.1% than that of CM at 12.6%. This aligns with the general consensus that SCM is 15–40 times more frequent than CM and persists for a longer duration (Seegers et al. 2003). The farmers’ lack of awareness of SCM, due to its subtle symptoms, which hinder early diagnosis and treatment, may contribute to its high prevalence.

The prevalence of Gram-negative bacilli associated with mastitis among cows in this study was 65.5%, with 75.7% in clinical cases and 62.7% in subclinical cases. These results were higher than those reported earlier by Tartor et al. (2021), who found a lower prevalence in the clinical cases (34.29%) and the subclinical cases (20%). This study shows that the prevalence of *Escherichia* spp. was 44%, while *Klebsiella* spp. was 23.1%, and *Pseudomonas* spp. was 11.6%. These findings are higher than the results reported by Lamey et al. (2026) (19%, 8%, and 7%, respectively). However, acute mastitis caused by *Klebsiella pneumoniae* is more severe and tends to respond less robustly to antibiotics than that caused by *E. coli*, leading to further decreased milk production, culling, or death (Emon et al. 2024).

*Pseudomonas* spp. isolates were detected in 11.6% of the total milk samples collected, whereas Mallick et al. (2025) reported a higher isolation rate of 42.22%. *Pseudomonas* infections pose a management problem due to their prolonged lifespan, and a single strain can be responsible for an entire outbreak on a dairy farm (Schauer et al. 2021).

Our results indicated that cows in their second and third parity have an increased risk of developing SCM compared to primiparous cows. This could mainly be due to udder and teat structural changes, immune system deterioration, a previous history of mastitis, and repeated exposure to milking processes (Gantner et al. 2024).

However, our study also revealed that cows with parity 4 or higher do not have a significantly higher risk of SCM than primiparous cows. Additionally, cows in their second parity have a lower risk of developing CM compared to primiparous cows. This may be attributed to several factors observed on some of the farms we visited, including poor mastitis detection, milking without distinguishing between healthy and mastitic cows, and restraining primiparous cows before milking, which may cause stress and injuries. Some farmers also reported observing teat self-sucking behaviour in cows. These factors were considered risks for mastitis in the primiparous dairy cows in the study by Persson Waller et al. (2021), who also found that milking primiparous cows at the calving site during the colostrum period was associated with CM.

Animals raised on open farms with frequent visitor contact, where visitors may select cows for purchase, have a significantly increased risk of developing SCM compared to those on farms with limited visitor contact. This may be explained by the potential introduction of pathogens through poor visitor hygiene, which negatively affects the farm’s hygiene. Additionally, stress caused by changes in routine can suppress cows’ immune systems.

We also found that the prevalence of SCM increased with the daily milk yield. Similar results were reported by Wang et al. (2024) (OR = 0.59; 95% CI: 0.419–0.849), who noted a relationship between high milk production and the duration the teat canal remains open, potentially increasing the risk of environmental pathogens invading the udder. Moreover, discarding the foremilk before milking is a practice associated with a reduced incidence of SCM (OR = 0.59; 95% CI: 0.39–0.89); this prevents high concentrations of bacteria from entering the milking machine and reduces the subsequent contamination of the udders by the machine (Bouchoucha et al. 2023).

This research reveals that a history of mastitis significantly increased CM prevalence. This finding is consistent with the study by Miyata et al. (2024), who reported that cows with a previous udder infection were more likely to be re-infected due to repeated challenges to the mammary tissues and/or the limited efficacy of treatment in eradicating the pathogens.

The presence of injury on the udder or teat showed a statistically significant association with the occurrence of CM. Such injuries are often due to mechanical factors resulting from non-compliance with milking techniques, technology, and the preparation of milking equipment, which may negatively affect the udder or teats (Andreeva et al. 2024).

The udder and leg hygiene were other risk factors identified in our study that increased the prevalence of CM. This finding agrees with the study by Rowe et al. (2021), who reported an incidence rate ratio (IRR) of 1.4 for each 10% increase in the proportion of cows with poor udder hygiene. This reflects the farmers’ awareness of prevention measures, as well as the poor hygiene conditions in dairy farms (Abebe et al. 2016).

Although this study did not identify any variables significantly associated with Gram-negative bacteria isolated from mastitic cows, Abegewi et al. (2022) reported a significant increase in vulnerability to coliform mastitis associated with the early lactation stage, a previous history of mastitis, breed, and muddy or wet faeces in an unclean environment. They stated that coliform mastitis was 5.9 times more prevalent in unclean environments than in clean ones. Balcha et al. (2022) also reported that the farm management and housing hygiene were significantly associated with the prevalence of *Escherichia coli* isolated from mastitic cows. Gram-negative bacteria are commonly found in manure, soil, bedding, water, and plant material. Poor hygiene can make the environment a reservoir for Gram-negative bacteria, increasing the risk of infection during humid, hot weather, which can lead to mastitis in dairy cows (Adkins et al. 2017).

In conclusion, the present study has demonstrated a high prevalence of mastitis, particularly the SCM, on dairy farms in Algeria. Both primiparous and multiparous cows with high milk production, poor scores for udder and leg hygiene, udder injuries, and a previous history of mastitis were at greater risk of developing mastitis. Furthermore, discarding the first stream of milk and restricting frequent contact with visitors were observed to reduce the risk of mastitis in cows.

The study also revealed that Gram-negative bacilli were isolated from more than half of the tested mastitic cows (65.5%), with *Escherichia* spp. being the most prevalent, followed by *Klebsiella* spp. and *Pseudomonas* spp.

These findings underscore the importance of developing strategic plans for the treatment, prevention, and control of mastitis in Algeria, alongside the necessity for further research to assess the phenotypic and molecular resistance profiles of the isolated bacteria.

## Supplementary Files

Electronic Supplementary Material (ESM) Tables
