# Supplementary material for: Bovine mastitis: Risk factors and isolation of Gram-negative bacteria in Western Algeria
Source: Vet Med (Praha). 2026 Feb 26;71(2):54–61. doi: 10.17221/40/2025-VETMED (PMC13004051; doi:10.17221/40/2025-VETMED)
Supplement: Electronic Supplementary Material (ESM) Tables [file VETMED-71-02-125040-s001.pdf]

https://doi.org/10.17221/40/2025-VETMED

Bovine mastitis: Risk factors and isolation of Gram-negative bacteria in Western Algeria

Cheimaa Bounoua[1], *Djahida Souna*[1], *Mohammed El Amine Bekara*[1], *Ibrahim Belabdi*[2], *Mohammed Sebaihia*[1], *Nora Mimoune*[3]*

[1]*Laboratory of Molecular Biology, Genomic and Bioinformatic, Department of Biology, Faculty of Nature and Life Sciences, University Hassiba Benbouali of Chlef, Chlef, Algeria*

[2]*Department of Biology, Faculty of Nature and Life Sciences, University Hassiba Benbouali of Chlef, Chlef, Algeria*

[3]*Higher National Veterinary School, Algiers, Algeria; Biotechnologies Platform for Animal Medicine & Reproduction (BIOMERA), Saad Dahleb Blida University 1, Blida, Algeria*

**Corresponding author: nora.mimoune@gmail.com*

The authors are fully responsible for both the content and the formal aspects of the electronic supplementary material. No editorial adjustments were made.

Electronic Supplementary Material (ESM)

Table S1. Descriptive statistics and univariate logistic regression analyses of the association of mastitis and Gram negative bacilli with different risk factors

Veterinarni Medicina, 71, 2026 (2): 54–61

https://doi.org/10.17221/40/2025-VETMED

Table S1. Descriptive statistics and univariate logistic regression analyses of the association of mastitis and Gram-negative bacilli with different risk factors

Risk factors	Category	Total examined	CMT results n (%)		P-value	Clinical mastitis n (%)		P-value	Gram-negative bacilli prevalence (%)		P-value
			positive n (%)	negative n (%)		positive n (%)	negative n (%)		positive n (%)	negative n (%)	
	<27	54 (10.3)	24 (44)	30 (56)		7 (13)	47 (87)		22 (71)	9 (29)	
Herd size	27–57	115 (21.9)	50 (43)	65 (57)	0.566 224	10 (9)	105 (91)	0.356 050	29 (48)	31 (52)	0.007 808*
	>57	355 (67.7)	173 (49)	182 (51)		49 (14)	306 (86)		154 (69)	68 (31)	
Control of newly arrived animals	yes	390 (74.4)	181 (46)	209 (54)	0.823 354	55 (14)	335 (86)	0.009 155*	170 (72)	66 (28)	0.112 546 2*
	no	112 (21.4)	50 (45)	62 (55)		5 (4)	107 (96)		33 (60)	22 (40)	
Type of farming	intensive	292 (55.7)	139 (48)	153 (52)		53 (18)	239 (82)		126 (66)	66 (34)	
	semi intensive	184 (35.1)	98 (53)	86 (47)	0.000 315*	13 (7)	171 (93)	<0.000 1*	71 (64)	40 (36)	0.592 276
	extensive	48 (9.2)	10 (21)	38 (79)		0 (0)	48 (100)		8 (80)	2 (20)	
	no	81 (15.5)	42 (52)	39 (48)		0 (0)	81 (100)		34 (81)	8 (19)	
Dry cow therapy	yes, systematically	240 (45.8)	103 (43)	137 (57)	0.199 316*	31 (13)	209 (87)	0.000 394*	93 (69)	41 (31)	0.007 488*
	yes, targeted	203 (38.7)	102 (50)	101 (50)		35 (17)	168 (83)		178 (57)	59 (43)	
	breeze block	468 (89.3)	229 (49)	239 (51)		61 (13)	407 (87)		185 (64)	105 (36)	
Construction materials	brick	30 (5.7)	13 (43)	17 (57)	0.011 650*	5 (17)	25 (83)	0.117 640 4*	16 (89)	2 (11)	0.592 276
	others	26 (5)	5 (19)	21 (81)		0 (0)	26 (100)		4 (80)	1 (20)	
	clean	198 (37.8)	87 (44)	111 (56)		16 (8)	182 (92)		78 (76)	25 (24)	
Stabe hygiene	middle	231 (44.1)	114 (49)	117 (51)	0.514 379	35 (15)	196 (85)	0.051 896*	84 (56)	65 (44)	0.004 233*
	poor	95 (18.1)	46 (48)	49 (52)		15 (16)	80 (84)		43 (70)	18 (30)	
	biocide	337 (64.3)	156 (46)	181 (54)		54 (16)	283 (84)		149 (71)	61 (29)	
Cleaning solution	soap	11 (2.1)	1 (9)	10 (91)	0.022 186*	0 (0)	11 (100)	0.004 883*	0 (0)	1 (100)	0.004 012*
	water	176 (33.6)	90 (51)	86 (49)		12 (7)	164 (93)		56 (55)	46 (45)	
Litter	yes	355 (67.7)	159 (45)	196 (55)	0.142 271*	52 (15)	303 (85)	0.055 945*	125 (59)	86 (41)	0.001 279*
	no	169 (32.3)	88 (52)	81 (48)		14 (8)	155 (92)		80 (78)	22 (22)	
Boot bath	yes	34 (6.5)	15 (44)	19 (56)	0.851 556	5 (15)	29 (85)	0.907 425	17 (85)	3 (15)	0.098 247*
	no	490 (93.5)	232 (47)	258 (53)		61 (12)	429 (88)		188 (64)	105 (36)	

https://doi.org/10.17221/40/2025-VETMED

Table S1 to be continued

Risk factors	Category	Total examined	CMT results n (%)		P-value	Clinical mastitis n (%)		P-value	Gram-negative bacilli prevalence (%)		P-value
			positive n (%)	negative n (%)		positive n (%)	negative n (%)		positive n (%)	negative n (%)	
Type of litter	straw	324 (61.8)	153 (47)	171 (53)	0.003 598*	52 (15)	303 (85)	0.004 44*	122 (60)	83 (40)	0.001 279*
	sawdust	31 (5.9)	6 (19)	25 (81)		14 (8)	155 (92)		3 (50)	3 (50)	
Litter renewal	no	175 (33.4)	87 (50)	88 (50)	0.567 760	16 (9)	159 (91)	0.000 749*	79 (77)	24 (23)	0.001 200*
	everyday	205 (39.1)	97 (47)	108 (53)		19 (9)	186 (91)		62 (53)	54 (47)	
	every week	144 (27.5)	63 (44)	81 (56)		31 (22)	113 (78)		64 (68)	30 (32)	
Milking method	manual	16 (3.1)	8 (50)	8 (50)	0.999 999	0 (0)	16 (100)	0.241 944*	6 (75)	2 (25)	0.719 108
	mechanical	508 (96.9)	239 (47)	269 (53)		66 (13)	442 (87)		199 (65)	106 (35)	
Milking clothing	yes	208 (39.7)	100 (48)	108 (52)	0.794 778	37 (18)	171 (82)	0.005 568*	98 (72)	39 (28)	0.062 518*
	no	316 (60.3)	147 (47)	169 (53)		29 (9)	287 (91)		107 (61)	69 (39)	
Presence of milking parlour	yes	445 (84.9)	209 (47)	236 (53)	0.949 014	52 (12)	393 (88)	0.191 514*	170 (65)	91 (35)	0.887 589
	no	79 (15.1)	38 (48)	41 (52)		14 (18)	65 (82)		35 (67)	17 (33)	
Hand prewashing	yes	337 (64.3)	149 (44)	188 (56)	0.087 535*	21 (11)	166 (89)	0.572 525	120 (62)	74 (38)	0.108 064*
	no	187 (35.7)	98 (52)	89 (48)		45 (13)	292 (87)		85 (71)	34 (29)	
Using milking gloves	yes	33 (6.3)	15 (45)	18 (55)	0.984 092	5 (15)	28 (85)	0.852 302	19 (95)	1 (5)	0.008 646*
	no	491 (93.7)	232 (47)	259 (53)		61 (12)	430 (88)		186 (63)	107 (37)	
Cleaning equipment	yes	451 (86.1)	209 (46)	242 (54)	0.434 883	55 (12)	396 (88)	0.619 664	166 (63)	98 (37)	0.036 033 0*
	no	73 (13.9)	38 (52)	35 (48)		11 (15)	62 (85)		39 (80)	10 (20)	
Teat probing	yes	313 (59.7)	140 (45)	173 (55)	0.209 029*	43 (14)	270 (86)	0.408 876	113 (62)	70 (38)	0.125 112*
	no	211 (40.3)	107 (50)	107 (50)		23 (11)	188 (89)		92 (71)	38 (29)	
Washing udder prior to milking	systematically	467 (90.8)	224 (47)	252 (53)	0.209 029*	62 (13)	414 (87)	0.837 962	186 (65)	100 (35)	0.701 574
	sometimes when it's dirty	42 (8)	21 (50)	21 (50)		4 (10)	38 (90)		18 (72)	7 (28)	
	no	6 (1.1)	2 (33)	4 (67)		0 (0)	6 (100)		1 (50)	1 (50)	

Veterinarni Medicina, 71, 2026 (2): 54–61

https://doi.org/10.17221/40/2025-VETMED

Table S1 to be continued

Risk factors	Category	Total examined	CMT results n (%)		P-value	Clinical mastitis n (%)		P-value	Gram-negative bacilli prevalence (%)		P-value
			positive n (%)	negative n (%)		positive n (%)	negative n (%)		positive n (%)	negative n (%)	
Drying of udder after washing	collective wipping	162 (30.9)	80 (49.4)	82 (50.6)		32 (23)	110 (77)		61 (59)	42 (41)	
	without wipping	362 (69.1)	167 (46.1)	195 (53.9)	0.482 738	34 (9)	348 (91)	<0.000 1*	144 (69)	66 (31)	0.131 510*
	individual wipping	0 (0)	0 (0)	0 (0)		0 (0)	0 (0)		0 (0)	0 (0)	
Dropping of milking clusters	yes	92 (17.6)	33 (36)	59 (64)	0.023 929*	20 (22)	72 (78)	0.009 704*	37 (70)	16 (30)	0.542 392
	no	416 (79.4)	206 (50)	210 (50)		46 (11)	370 (89)		162 (64)	90 (36)	
Visitors contacting cattle	yes	58 (11.1)	37 (64)	21 (36)	0.010 615*	10 (17)	48 (83)	0.357 059 0	24 (51)	23 (49)	0.036 513*
	no	466 (88.9)	210 (45)	256 (55)		56 (12)	410 (88)		181 (68)	85 (32)	
Elimination of the first jets	yes ,under the animal	294 (56.1)	129 (44)	165 (56)		39 (13)	255 (87)		99 (59)	69 (41)	
	no	230 (43.9)	118 (51)	112 (49)	0.109 168*	27 (12)	203 (88)	0.696 636	106 (73)	39 (27)	0.012 028*
	yes, in the recipient	0 (0)	0 (0)	0 (0)		0 (0)	0 (0)		0 (0)	0 (0)	
Frequency of veterinary visits	frequent	227 (43.3)	113 (50)	114 (50)		18 (8)	209 (92)		83 (63)	48 (37)	
	very frequent	146 (27.6)	61 (42)	85 (58)	0.300 366	33 (23)	113 (77)	<0.000 1*	58 (62)	36 (38)	0.234 607*
	rarely	151 (28.8)	73 (48)	78 (52)		15 (10)	136 (90)		64 (73)	24 (27)	
Veterinary hygiene	yes	113 (21.6)	45 (40)	68 (60)	0.098 461*	19 (17)	94 (83)	0.171 917*	36 (56)	28 (44)	0.110 276*
	no	411 (78.4)	202 (49)	209 (51)		47 (11)	364 (89)		169 (68)	80 (32)	
Separation of sick cows	yes	321 (61.3)	152 (47)	169 (53)	0.972 924	51 (16)	270 (84)	0.006 503*	133 (66)	70 (34)	1
	no	203 (38.7)	95 (47)	108 (53)		15 (7)	188 (93)		72 (65)	38 (35)	
History of abortion in the herd	yes	286 (54.6)	136 (48)	150 (52)	0.903 884	50 (17)	236 (83)	0.000 365*	107 (58)	79 (42)	0.000 524*
	no	238 (45.4)	111 (47)	127 (53)		16 (7)	222 (93)		98 (77)	29 (23)	
Abortion of mastitic cows	yes	113 (21.6)	56 (50)	57 (50)	0.634 412	20 (18)	93 (82)	0.091 756*	44 (58)	32 (42)	0.143 433*
	no	411 (78.4)	191 (46)	220 (54)		22 (7)	274 (93)		161 (68)	76 (32)	

https://doi.org/10.17221/40/2025-VETMED

Table S1 to be continued

Risk factors	Category	Total examined	CMT results n (%)		P-value	Clinical mastitis n (%)		P-value	Gram-negative bacilli prevalence (%)		P-value
			positive n (%)	negative n (%)		positive n (%)	negative n (%)		positive n (%)	negative n (%)	
Veterinary consultation	yes	228 (43.5)	101 (44)	127 (56)	0.291 696	44 (19)	184 (81)	<0.000 1*	86 (59)	59 (41)	0.043 469*
	no	296 (56.5)	146 (49)	150 (51)		22 (7)	274 (93)		119 (71)	49 (29)	
History of infertility in the herd	yes	240 (45.8)	102 (42)	138 (58)	0.061 887*	38 (16)	202 (84)	0.054 679*	75 (54)	65 (46)	0.000 107*
	no	284 (54.2)	145 (51)	139 (49)		28 (10)	256 (90)		130 (75)	43 (25)	
Tick infestation	yes	245 (46.8)	111 (45)	134 (55)	0.484 397	47 (19)	198 (81)	<0.000 1*	94 (59)	64 (41)	0.032 669*
	no	279 (53.2)	136 (49)	143 (51)		19 (7)	260 (93)		111 (72)	44 (28)	
Season	summer	385 (73.5)	174 (45)	211 (55)	0.166 525*	43 (11)	342 (89)	0.136 517 8*	156 (72)	61 (28)	0.000 563*
	winter	139 (26.5)	73 (53)	66 (47)		23 (17)	116 (83)		49 (51)	47 (49)	
Province	Chlef	109 (20.3)	45 (41.3)	64 (58.7)	0.093*	26 (23.9)	83 (76.1)	0.000*	56 (78.9)	15 (21.1)	0.000*
	Ain defla	83 (15.8)	49 (59)	34 (41)		16 (19.3)	67 (80.7)		28 (43.1)	37 (56.9)	
	Sidi bel abbes	196 (37.4)	89 (45.4)	107 (54.6)		6 (3.1)	190 (96.9)		68 (71.6)	27 (28.4)	
	Relizane	136 (26)	64 (47.1)	72 (52.9)		18 (13.2)	118 (86.8)		53 (64.6)	29 (35.4)	
Cow breed	Holstein	360 (68.7)	162 (45)	198 (55)	0.071 011*	52 (14)	308 (86)	0.167 348*	141 (66)	73 (34)	0.958 359
	Montbeliarde	130 (24.8)	72 (55)	58 (45)		11 (9)	119 (92)		54 (65)	29 (35)	
	others	34 (6.5)	13 (38)	21 (62)		3 (9)	31 (91)		10 (62)	6 (38)	
Age (year)	2–5	374 (71.4)	182 (49)	192 (51)	0.013 347*	47 (13)	327 (87)	0.167 348*	151 (66)	78 (34)	0.958 359
	6–9	145 (27.7)	60 (41)	85 (59)		19 (13)	126 (87)		52 (66)	27 (34)	
	>9	5 (1)	5 (100)	0 (0)		0 (0)	5 (100)		2 (40)	3 (60)	
Parity	1	148 (28.2)	53 (36)	95 (64)	0.008 027*	25 (17)	123 (83)	0.049 108*	59 (76)	19 (24)	0.088 096*
	2	171 (32.6)	92 (54)	79 (46)		12 (7)	159 (93)		63 (61)	41 (39)	
	3	97 (18.5)	51 (53)	46 (47)		13 (13)	84 (87)		44 (69)	20 (31)	
	≥4	108 (20.6)	51 (47)	57 (53)		16 (15)	92 (85)		39 (58)	28 (42)	
Stage of lactation	1 month	104 (19.8)	50 (48)	54 (52)	0.939 405	10 (10)	94 (90)	0.578 914	45 (75)	15 (25)	0.176 287*
	2–4 months	212 (40.5)	98 (46)	114 (54)		29 (14)	183 (86)		83 (65)	44 (35)	
	≥5 months	208 (39.7)	99 (48)	109 (52)		27 (13)	181 (87)		77 (61)	49 (39)	

Original Paper
Veterinarni Medicina, 71, 2026 (2): 54–61
https://doi.org/10.17221/40/2025-VETMED

Table S1 to be continued

Risk factors	Category	Total examined	CMT results *n* (%)		*P*-value	Clinical mastitis *n* (%)		*P*-value	Gram-negative bacilli prevalence (%)		*P*-value
			positive *n* (%)	negative *n* (%)		positive *n* (%)	negative *n* (%)		positive *n* (%)	negative *n* (%)	
Milk production/day	<5 l	14 (2.7)	2 (14)	12 (86)		0 (0)	14 (100)		0 (0)	2 (100)	
	5–10 l	467 (89.1)	222 (49)	243 (51)	0.044 112*	4 (9)	39 (91)	0.345 677	186 (65)	100 (35)	0.087 504*
	>10 l	43 (8.2)	21 (48)	22 (52)		62 (13)	405 (87)		19 (76)	6 (24)	
Udder conformation defects	present	284 (54.2)	136 (48)	148 (52)	0.774 674	44 (15)	240 (85)	0.041 107*	113 (63)	67 (37)	0.290 854
	absent	240 (45.2)	129 (54)	111 (46)		22 (9)	218 (91)		92 (69)	41 (31)	
Height and balance	normal	432 (82.4)	197 (46)	235 (54)		47 (11)	385 (89)		169 (69)	75 (31)	
	udder too low	14 (2.7)	7 (50)	7 (50)	0.293 230 8	5 (36)	9 (64)	0.010 193*	3 (25)	9 (75)	0.002 884*
	udder badly balanced	78 (14.9)	43 (55)	35 (45)		14 (18)	64 (82)		33 (58)	24 (42)	
Quality of udder attachments	yes	517 (98.7)	243 (47)	274 (53)	0.878 597	64 (12)	453 (88)	0.216 540*	201 (65)	106 (35)	0.002 884*
	no	7 (1.3)	4 (57)	3 (43)		2 (29)	5 (71)		4 (67)	2 (33)	
Teats position	teats not aligned	133 (25.4)	59 (44)	74 (56)		21 (16)	112 (84)		48 (60)	32 (40)	
	weak suspensory ligament	64 (12.2)	39 (61)	25 (39)	0.059 995*	8 (12)	56 (88)	0.423 157	31 (66)	16 (34)	0.475 000
	normal	327 (62.4)	149 (46)	178 (54)		37 (11)	290 (89)		126 (68)	60 (32)	
Teat size	unequal	101 (19.3)	45 (45)	56 (55)		17 (17)	84 (83)		39 (63)	23 (37)	
	too short, too long, too thin or too thick	55 (10.5)	29 (53)	26 (47)	0.618 019	11 (20)	44 (80)	0.047 192*	22 (55)	18 (45)	0.241 632*
	normal	368 (70.2)	173 (47)	195 (53)		38 (10)	330 (90)		144 (68)	67 (32)	
Teat shape	conical	7 (1.3)	4 (57)	3 (43)		2 (29)	5 (71)		5 (83)	1 (17)	
	cylindrical	3 (0.6)	2 (67)	1 (33)	0.618 019	0 (0)	3 (100)	0.478 602	2 (100)	0 (0)	0.241 632
	normal	514 (98.1)	241 (47)	273 (53)		64 (12)	450 (88)		198 (65)	107 (35)	
Udder injuries	yes	11 (2.1)	2 (18)	9 (82)	0.101 183*	6 (55)	5 (45)	0.000 891*	6 (75)	2 (25)	0.719 108
	no	513 (97.9)	245 (48)	268 (52)		60 (12)	453 (88)		199 (65)	106 (35)	

https://doi.org/10.17221/40/2025-VETMED

Table S1 to be continued

Risk factors	Category	Total examined	CMT results n (%)		P-value	Clinical mastitis n (%)		P-value	Gram-negative bacilli prevalence (%)		P-value
			positive n (%)	negative n (%)		positive n (%)	negative n (%)		positive n (%)	negative n (%)	
Udder and leg hygiene score	1	151 (28.8)	71 (47)	80 (53)	0.260 386	5 (3)	146 (97)	<0.000 1*	54 (71)	22 (29)	0.672 039
	2	185 (35.3)	95 (52)	90 (48)		14 (8)	171 (92)		70 (64)	39 (36)	
	3	160 (30.5)	66 (41)	94 (59)		38 (24)	122 (76)		65 (62)	39 (38)	
	4	28 (5.3)	15 (54)	13 (46)		9 (32)	19 (68)		16 (67)	8 (33)	
History of mastitis	yes	108 (20.6)	61 (56)	47 (44)	0.037 976*	22 (20)	86 (80)	0.010 159*	45 (54)	38 (46)	0.016 997*
	no	416 (79.4)	186 (45)	230 (55)		44 (11)	372 (89)		160 (70)	70 (30)	
History of infertility	yes	61 (11.6)	22 (36)	39 (64)	0.087 920*	9 (15)	52 (85)	0.737 389	18 (58)	13 (42)	0.472 841
	no	463 (88.4)	225 (49)	238 (51)		57 (12)	406 (88)		187 (66)	95 (34)	
History of abortion	yes	11 (2.1)	3 (27)	8 (73)	0.303 627	2 (18)	9 (82)	0.637 030	4 (80)	1 (20)	0.472 841
	no	513 (97.9)	244 (48)	269 (52)		64 (12)	449 (88)		201 (65)	107 (35)	
Cow acquisition	buying	406 (77.5)	184 (45)	222 (55)	0.149 590*	54 (13)	352 (87)	0.456 457	164 (69)	74 (31)	0.033 756*
	breeding stock	118 (22.5)	63 (53)	55 (47)		12 (10)	106 (90)		41 (55)	34 (45)	

*P-value of variable effect
